# *Nesterenkonia* sp. strain F, a halophilic bacterium producing acetone, butanol, and ethanol under aerobic conditions

**DOI:** 10.1038/srep18408

**Published:** 2016-01-04

**Authors:** Hamid Amiri, Reza Azarbaijani, Laleh Parsa Yeganeh, Abolhassan Shahzadeh Fazeli, Meisam Tabatabaei, Ghasem Hosseini Salekdeh, Keikhosro Karimi

**Affiliations:** 1 Department of Biotechnology, Faculty of Advanced Sciences and Technologies, University of Isfahan, Isfahan 81746-73441, Iran; 2 Molecular Bank, Iranian Biological Resource Center, ACECR, Tehran, Iran; 3 Department of Molecular and Cellular Biology, Faculty of Basic Sciences and Advanced Technologies in Biology, University of Science and Culture, ACECR, Tehran, Iran; 4 Agricultural Biotechnology Research Institute of Iran (ABRII), AREEO, Karaj, Iran; 5 Biofuel Research Team (BRTeam), Karaj, Iran; 6 Industrial Biotechnology Group, Institute of Biotechnology and Bioengineering, Isfahan University of Technology, Isfahan 84156-83111, Iran; 7 Department of Chemical Engineering, Isfahan University of Technology, Isfahan, 84156-83111, Iran

## Abstract

The moderately halophilic bacterium *Nesterenkonia* sp. strain F, which was isolated from Aran-Bidgol Lake (Iran), has the ability to produce acetone, butanol, and ethanol (ABE) as well as acetic and butyric acids under aerobic and anaerobic conditions. This result is the first report of ABE production with a wild microorganism from a family other than Clostridia and also the first halophilic species shown to produce butanol under aerobic cultivation. The cultivation of *Nesterenkonia* sp. strain F under anaerobic conditions with 50 g/l of glucose for 72 h resulted in the production of 105 mg/l of butanol, 122 mg/l of acetone, 0.2 g/l of acetic acid, and 2.5 g/l of butyric acid. Furthermore, the strain was cultivated on media with different glucose concentrations (20, 50, and 80 g/l) under aerobic and anaerobic conditions. Through fermentation with a 50 g/l initial glucose concentration under aerobic conditions, 66 mg/l of butanol, 125 mg/l of acetone, 291 mg/l of ethanol, 5.9 g/l of acetic acid, and 1.2 g/l of butyric acid were produced. The enzymes pertaining to the fermentation pathway in the strain were compared with the enzymes of *Clostridium* spp., and the metabolic pathway of fermentation used by *Nesterenkonia* sp. strain F was investigated.

The volatility of the fossil resources supplying increasing worldwide demands for fuel and chemicals, as well as the environmental perturbation caused by their over-consumption of these resources, has become a crucial problem worldwide in this century. Biomass has gained increased attention as a renewable source for the production of liquid fuels and a variety of chemicals. Furthermore, to move toward a true low CO_2_ future, technologies for the production of bio-based products should be developed^1^,^2^.

Acetone, butanol, and ethanol (ABE) fermentation is an old fermentation process that has gained renewed interest in recent years, especially the production of butanol as an advanced biofuel^3^,^4^,^5^. Up to the 1950s, more than 60% of butanol and 10% of acetone worldwide were produced by ABE fermentation from corn starch and molasses^6^. However, the butanol and acetone production route was shifted to petrochemical-based processes after the 1980s. In recent years, different studies have been conducted to improve ABE fermentation as a bacterial branched chain pathway for obtaining ABE, as well as acetic acid and butyric acid, from renewable resources^7^.

All bacteria reported to date that are capable of naturally producing n-butanol belong to the Clostridia family^8^. Clostridia are strict anaerobes and grow relatively slowly compared with other well-characterized aerobic microorganisms, leading to lower productivity^9^. Simultaneous spore formation, low attainable cell densities during anaerobic fermentation, and poorly-characterized strain degeneracy are among the characteristics of fermentation by Clostridia that limit the yield of ABE production by these bacteria^9^. Furthermore, bacteriophages contamination is among the major problems of traditional ABE production processes, making continuous fermentation operations difficult. As a result, the rigorous sterilization of ABE production plants with high energy requirements is inevitable. Moreover, the production of a dilute product stream results in a high amount of fresh water consumption. Thus, there have been efforts to use alternative strains for utilizing renewable carbon sources for the production of solvents and acids. Efforts to overcome some of the problems associated with the clostridial butanol-production process have included the introduction of the clostridial fermentative pathway into heterologous organisms, e.g., *Escherichia coli*^10^,^11^, *Bacillus subtilis*^12^, and *Saccharomyces cerevisiae*^13^. In addition, Lan *et al.* reported photosynthetic n-butanol biosynthesis in cyanobacteria by replacing the oxygen-sensitive coenzyme A-acylating aldehyde dehydrogenase with oxygen-tolerant enzymes^14^. However, much more metabolic engineering and optimization effort is required to obtain a proper strain by the introduction of a clostridial fermentative pathway into a heterologous host^9^.

The sensitivity of Clostridia to high osmotic media is another drawback of these species in ABE production, as a number of inexpensive media, e.g., dilute acid hydrolysates of lignocelluloses and molasses, have high osmotic pressures^15^. From another point of view, using a halophile for ABE production may be a breakthrough in the development of low cost processes for the production of biochemical and biofuels. Halophiles have recently been recognized as advantageous microorganisms for use in bioprocesses^16^,^17^,^18^. For instance, halophiles are strong candidates for non-sterile and continuous bioprocessing using seawater as media^19^. As a result, halophile strains are promising candidates for the development of low cost and contamination-free continuous bioprocesses with low energy and fresh water consumptions.

*Nesterenkonia* was originally identified as an aerobic organic-solvent-tolerant and alpha amylase producing organism^1^,^20^,^21^. Recently, the publication of the genome sequence of *Nesterenkonia* sp. strain F^20^ revealed that the bacterium possessed a large number of carbohydrate-related genes, as well as genes involved in butanol fermentation and monosaccharide/polysaccharide utilization. However, to our knowledge, there are no reports of using this strain or similar halophiles for ABE production or acetic and butyric acid production.

In this study, the capabilities of a wild strain of a halophilic *Nesterenkonia* sp. were evaluated for solvent and acid production under aerobic, anaerobic, and halophilic conditions. The genome sequence was analyzed and related to the metabolites that were produced.

## Materials and Methods

### Fermentation

The bacterium *Nesterenkonia* sp. strain F which was isolated from Aran-Bidgol Salt Lake in Iran was comprehensively identified morphologically, physiologically and molecularly by Amoozegar *et al.*^22^ and was deposited in Iranian Biological Resource Center (IBRC) under the accession number IBRC-M 10223. The bacterium was cultivated both under aerobic and anaerobic conditions in 118 ml serum bottles containing 50 ml of culture medium consisting of the following (g/l): 81 NaCl, 7 MgCl_2_, 9.6 MgSO_4_, 0.36 CaCl_2_, 2 KCl, 0.06 NaHCO_3_, 0.026 NaBr, 5 proteose peptone, 10 yeast extract, and specific amounts of glucose (20 to 80 g/l). The medium was autoclaved at 121 °C for 20 min, and NaHCO_3_ was added from a sterilized stock solution after the medium was cooled to room temperature. Anaerobic conditions were generated by purging the bottles with pure nitrogen passed over a heated reduced copper column to remove trace oxygen. To maintain anaerobic conditions, the bottles were sealed with a butyl rubber stopper fastened with an aluminum crimp. Unless otherwise stated, the cultures were incubated at 32 °C for 72 h and samples were periodically withdrawn through the rubber stopper by a sterile syringe during fermentation. The samples were stored at −18 °C until analyses for the sugar and metabolites. The aerobic experiments were conducted similarly except that no nitrogen was purged into the bottles prior to inoculation.

### Analytical procedures

High-performance liquid chromatography (HPLC) equipped with UV/VIS and RI detectors (Agilent 1260 Infinity, USA) was used for the analysis of the metabolites. Acetone, butanol, ethanol, acetic acid, and butyric acid were analyzed using an Aminex HPX-87H column (Bio-Rad, Richmond, CA, USA) at 60 °C with 0.6 ml/min eluent of 0.005 M sulfuric acid. In addition, the glucose remaining after cultivation was determined using an Aminex HPX-87P column (Bio-Rad, Richmond, CA, USA) at 80 °C with deionized water as an eluent at a flow rate of 0.6 ml/min. The concentrations of glucose, acetone, butanol, and ethanol were determined by an RI detector, whereas acetic acid and butyric acid were quantified on UV chromatograms at 210 nm using the LC Chemstation software (Agilent, USA). Moreover, a gas chromatography (GC) (Agilent 6890, USA) equipped with an FID detector and an HP INNOwax capillary column (60 m × 0.32 mm i.d., 0.5 μm film thickness) was used to confirm the obtained results. Nitrogen was used as the carrier gas with a flow rate of 2 ml/min. The oven temperature was increased to 170 °C at a rate of 3 °C/min followed by a ramp of 10 °C/min to 250 °C. The oven temperature was held at 250 °C for 5 min. The injector and detector temperatures were also set at 250 °C.

### Genome sequence and phylogenetic analysis

Sarikhan *et al.* (2011) sequenced the genome of *Nesterenkonia* sp. strain F by using the 454 GS-FLX Titanium technology^20^. They conducted an enriched-annotation of the genome by using the rapid annotation using subsystem technology (RAST) server as previously described^23^. The 2,812,133 bp draft genome reportedly contained 2,484 genes, with 1,794 nonhypothetical and 690 hypothetical protein coding sequences and 50 structural RNAs. Moreover, function assigning and profiling of the presence and abundance of enzymes were analyzed using Integrated Microbial Genomes (IMG) tools^24^.

## Results and Discussion

### ABE fermentation under clostridial cultivation conditions

**Figure 1:**
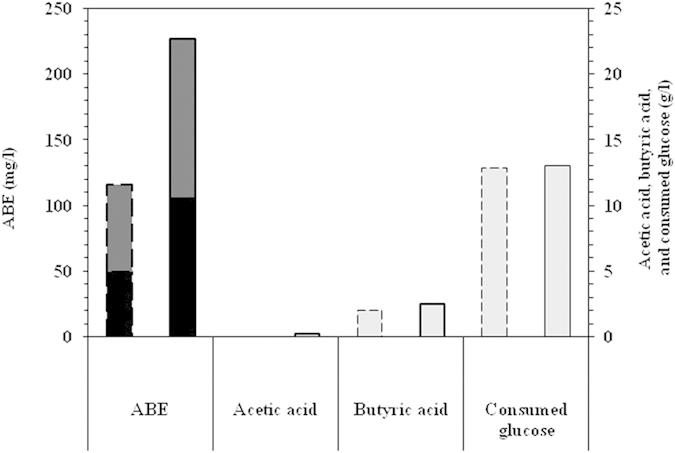
Concentrations of fermentation products, i.e., butanol (black), acetone (dark gray), ethanol (white), acetic acid, and butyric acid (light gray), as well as consumed glucose (light gray) detected through the fermentation of glucose with an initial concentration of 50 g/l at 37 °C under anaerobic conditions for 48 (dashed border) and 72 h (solid border).

The bacterium *Nesterenkonia* sp. strain F was cultivated under conditions conventionally used for ABE fermentation by Clostridia, i.e., anaerobic fermentation with 50 g/l of glucose supplemented with the required nutrients at 37 °C. Solvent and acid production, as well as a glucose consumption profile, are shown in Fig. 1. Cultivation under anaerobic conditions resulted in sugar consumption of up to 13 g/l after 48 h. During the 48 h cultivation, 49.7 mg/l of butanol, 66.7 of mg/l acetone, and 2.1 g/l of butyric acid were produced without acetic acid or ethanol production. After 48 h of fermentation, the concentration of glucose remained constant.

The final concentrations of the products after 72 h of fermentation increased to more than 105 mg/l of butanol, 122 mg/l of acetone, 0.2 g/l of acetic acid, and 2.5 g/l of butyric acid. Solventogenic Clostridia, e.g., *C. acetobutylicum* 260, *C. acetobutylicum* 824, *C. saccharobutylicum* 262, and *C. butylicum* 592, reportedly produce more than 16 g/l of total ABE in media containing 60 g/l glucose under anaerobic conditions^25^. Although the total ABE concentration produced by the *Nesterenkonia* sp. strain F was lower than the concentration produced by Clostridia, the butyric acid concentration produced by this strain was higher than that reportedly produced by Clostridia.

The cultivation of *Nesterenkonia* sp. strain F at 37 °C under anaerobic conditions conventionally used for ABE fermentation resulted in the fermentative conversion of glucose to 105 mg/l of butanol along with other products. Although the optimal growth of this strain was previously reported at 32 °C under aerobic conditions, the strain was grown herein in relatively different conditions^22^. In the draft genome sequence analyses, this strain was shown to contain the genes responsible for resistance to oxidative and heat shock stresses making it capable of growing at elevated temperatures and under anaerobic conditions^20^. Because the fermentation products were considered the main objective of the present study, the optimal conditions for the production of these desired products may not necessarily have been identical to those required for maximum growth. Hence, the strain was cultivated under different conditions to investigate the production of ABE, as well as acetic acid and butyric acid (cf. *ABE fermentation under varying cultivation conditions*).

### ABE fermentation under varying cultivation conditions

**Figure 2:**
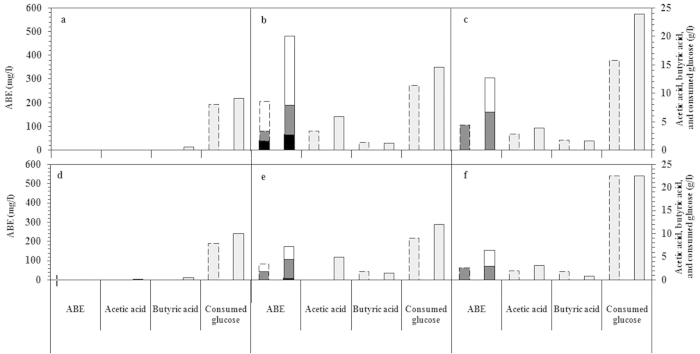
Concentrations of fermentation products, i.e., butanol (black), acetone (dark gray), ethanol (white), acetic acid, and butyric acid (light gray), as well as consumed glucose (light gray) obtained through fermentation of glucose with initial concentration of 20 (a,d), 50 (b,e), and 80 g/l (c,f) at 32 °C under aerobic (a–c) and anaerobic conditions (d–f) for 48 (dashed border) and 72 h (solid border).

The strain was cultivated under aerobic and anaerobic conditions in media containing 20, 50, or 80 g/l of glucose. The consumption of glucose and the production of acids and solvents through fermentation are depicted in Fig. 2. As shown, the cultivation of the bacteria resulted in the production of different amounts of products depending on the conditions applied.

These results showed that glucose consumption as well as solvent and acid formation, was greatly affected by the concentration of glucose, as well as the concentration of oxygen, in the media. Depending on the cultivation conditions, 9–24 g/l of glucose were consumed by the microorganism, leading to different amounts of ABE, from less than 1 to 482 mg/l. The production of acetone, butanol, ethanol, acetic acid and butyric acid by branched fermentation is a strategy to optimize energy production under anaerobic conditions as an energy-stressed situations^26^. As shown in Fig. 2, even in the presence of oxygen, the *Nesterenkonia* sp. strain F fermented glucose to products, i.e., 482 mg/l of ABE and 7.1 g/l of acid. Although fermentation is mostly used by organisms to adapt to anoxic environments, it is not confined to such conditions^27^.

As shown in Fig. 2, the *Nesterenkonia* sp. strain F produced higher amounts of solvents and acids under aerobic conditions than under anaerobic conditions. More specifically, in this study, aerobic cultivation of bacteria resulted in 195% higher solvent and 13% higher acid production compared with anaerobic conditions (initial glucose concentration of 50 g/l). As an aerobic bacteria, *Nesterenkonia* sp. strain F’s catabolism and anabolism are based mainly on oxidative pathways; therefore, *Nesterenkonia* sp. strain F has inefficient growth and activity under anaerobic conditions^28^. Therefore, the reduction of the ABE concentration from 482 mg/l to 174 mg/l in the absence of oxygen could be ascribed to inefficient microbial growth. In addition, oxidative and fermentative metabolism may be more interrelated in bacteria than they are in yeasts^27^. Using an initial glucose concentration of 20 g/l, more than 45% of the glucose content of the medium was consumed through oxidative metabolism, leaving a minor amount of glucose for fermentative pathways. This low level led to negligible production of ABE and acids, even under aerobic conditions. Mixed-acid and solvent fermentation by bacteria are totally different from yeast fermentation, in which acetyl-CoA is both an intermediate in the TCA cycle of aerobic oxidation of pyruvate and a key intermediate for acid and solvent fermentation in bacteria, e.g., *C. acetobutylicum*, *E. coli*, and *Nesterenkonia*. Therefore, unlike ethanol fermentation, which converts pyruvate to ethanol independent of aerobic oxidation pathways, mixed acid fermentation pathways contain a part of the aerobic pathway, the conversion of pyruvate to acetyl-CoA, and may be dependent on aerobic oxidation. The partial supplying of the catabolic requirements of cells by fermentative and oxidative metabolism has been proven for *E. coli*^27^,^29^. For the selection of partial metabolism by *E. coli*, different interrelated causes are involved, including the inequality of glucose metabolism and respiration, the high influx of carbon into the cell exceeding the demands for biosynthesis, the presence of excess NADH, the repression of TCA cycle enzymes, and the uncoupling of metabolism^29^.

By contrast, the halophilic characteristics of *Nesterenkonia* may reduce the capacity of the oxidative pathway, leading to use of a partial pathway. In fact, *Nesterenkonia* sp. strain F is a halophile that grows in high concentrations of NaCl up to 200 g/l^30^. The oxygen equilibrium concentration in the media containing 81 g/l NaCl, which was used for the cultivation of *Nesterenkonia* sp. strain F, was reported to be less than 4.7 mg/l, 35% lower than the equilibrium oxygen concentration in pure water^31^. Therefore, the limited capacity of oxidative metabolism as a consequence of low oxygen concentrations in media may be responsible for aerobic branched fermentation by the *Nesterenkonia* strain F as observed in the present study. This finding was in line with the findings of Han *et al.* who highlighted the limited capacity of oxidative metabolism in *E. coli* as one of the main reasons responsible for aerobic fermentation^27^.

The aerobic fermentation and oxidative metabolism of *Nesterenkonia* sp. strain F were greatly affected by the initial glucose concentration. Through the cultivation of *Nesterenkonia* in a medium with 20 g/l glucose, more than 9 g/l glucose were consumed without the formation of any detectable fermentative products, revealing that glucose carbon flux was mostly directed into the oxidative metabolism of the cell. Increasing the initial glucose concentration to 50 g/l resulted in the formation of relatively higher amounts of total solvents and acids as a result of carbon flow into fermentation. Therefore, aerobic fermentation by *Nesterenkonia* sp. strain F in media containing 50 g/l of glucose resulted in the formation of 5.9 g/l of acetic acid, 1.2 g/l of butyric acid, 291 mg/l of ethanol, 125 mg/l of acetone, and 66 mg/l of butanol after 72 h. Increasing the initial glucose concentration from 50 to 80 g/l resulted in an approximately 37% reduction in total ABE and 21% in total acid formation after 72h of fermentation. Reductions in product concentrations at high sugar concentrations has also been reported for *E. coli*^29^, and *Clostridium* spp.^32^, due to product inhibition. In ABE fermentation without product removal by strains of solventogenic Clostridia, e.g., *C. acetobutylicum* and *C. beijerinckii*, sugar concentrations higher than 60 g/l were not recommended due to product inhibition caused by butanol^33^. In addition, *E. coli* strains, i.e., JM105, B, W3110, W3100, HB101, DH1, CSH50, MC1060, JRG1046, and JRG1061, were reportedly inhibited by acetic acid as a fermentation product^29^.

As shown in Fig. 2, in addition to acids, solvents, i.e., ABE, were produced through the cultivation of *Nesterenkonia* sp. strain F in media with more than 50 g/l of glucose. Therefore, natural formation of butanol, which has been considered as exclusive to the Clostridia family^8^, was also observed in *Nesterenkonia* sp. strain F, a halophile bacterium of the *Micrococcaceae* family of the Actinomycetales order^34^.

Clostridia are strictly anaerobic bacteria and produce butanol only under anaerobic conditions. Considering the difficulties of butanol production by Clostridia, e.g., providing strictly anaerobic conditions, much effort has been expended to obtain a modified strain for biobutanol production. An engineered *E. coli* with the ability to produce butanol has been obtained by transferring an essential set of genes from *C. acetobutylicum* and overexpressing acetyl-CoA acetyltransferase from *E. coli*^10^. Aerobic cultivation of the engineered *E. coli* at 37 °C for 40 h resulted in less than 10 mg/l of butanol production^10^. In addition, metabolic engineering of *S. cerevisiae* has been used for butanol production in semi-aerobic conditions^13^; however, its cultivation resulted in the production of less than 2.5 mg/l of butanol from media with 20 g/l of galactose^13^. Thus, compared with the reported modified strains, the *Nesterenkonia* sp. strain F seems to be a promising platform for ABE production under aerobic conditions. More promising results could be achieved if this strain would be subjected to genetic modifications.

### Genome-wide overview of carbohydrate active enzymes for *Nesterenkonia* sp. strain F

**Figure 3:**
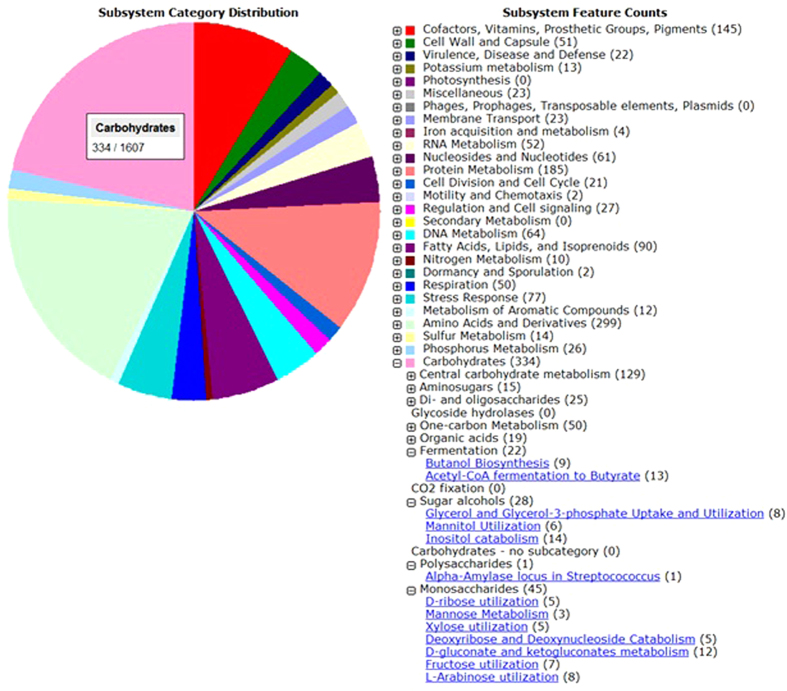
Subsystem category distribution of *Nesterenkonia* sp. strain F encoding genes.

The draft genome analysis of *Nesterenkonia* sp. strain F showed that this strain is a rich source of carbohydrate active enzymes. Genome annotation using RAST tools revealed that 20.8% of the subsystem categorized genes were classified as being involved in carbohydrate metabolism (Fig. 3). The genes encoding carbohydrate active enzymes were grouped based on enzyme families (Table 1) and their substrate specificity (Table 2) compared with the only draft genome reported for *Nesterenkonia* spp., i.e., *Nesterenkonia alba* DSM 19423. This comparative genome study illustrated the superior ability of *Nesterenkonia* sp. strain F compared with *Nesterenkonia alba* DSM 19423 to catabolize oligosaccharides and polysaccharides, owing to a higher variety and quantity of sugar hydrolysis encoding genes.

### Enzymes involved in ABE production by *Nesterenkonia* sp. strain F

**Figure 4:**
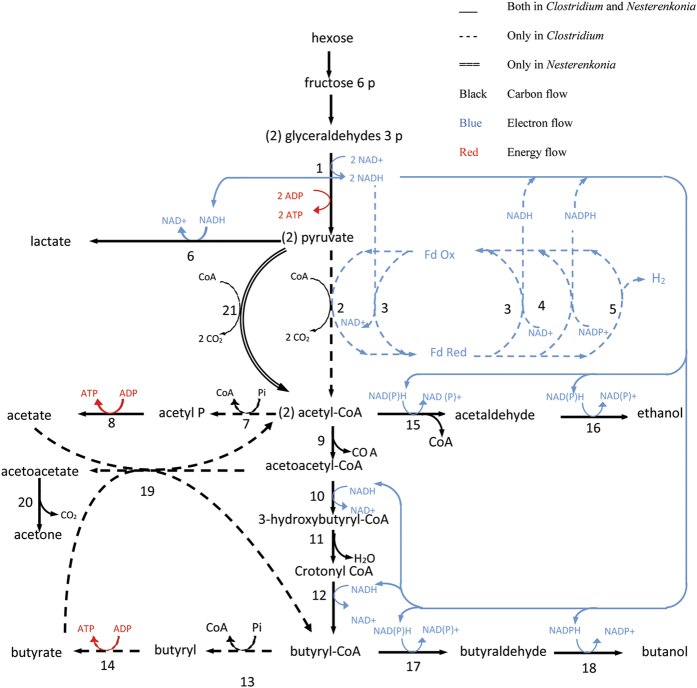
Catabolic pathways used by solvent-producing Clostridia. The directions of carbon and electron flow are shown by black and blue arrows, respectively. Enzymes indicated by numbers are as follows: (1) glyceraldehyde-3-phosphate dehydrogenase, (2) pyruvate-ferredoxin oxidoreductase, (3) NADH-ferredoxin oxidoreductase, (4) NADPH-ferredoxin oxidoreductase, (5) hydrogenase, (6) lactate dehydrogenase, (7) phosphate acetyltransferase (phosphotransacetylase), (8) acetate kinase, (9) thiolase (acetyl-CoA acetyltransferase), (10) 3-hydroxybutyryl-CoA dehydrogenase, (11) crotonase, (12) butyryl-CoA dehydrogenase, (13) phosphate butyltransferase (phosphotransbutyrylase), (14) butyrate kinase, (15) acetaldehyde dehydrogenase, (16) ethanol dehydrogenase, (17) butyraldehyde dehydrogenase, (18) butanol dehydrogenase, (19) acetoacetyl-CoA: acetate/butyrate: CoA transferase, (20) acetoacetate decarboxylase, and (21) pyruvate dehydrogenase.

Through cultivation of the moderate halophile strain *Nesterenkonia* sp. strain F solvents (i.e., ABE) and acids (i.e., acetic and butyric acids) were produced. The enzymes pertaining to the ABE fermentation pathway in *Nesterenkonia* sp. strain F are shown in Table 3, and are compared with those of *Clostridium* spp.^26^. Based on the presence of these enzymes, the apparent metabolic pathways of fermentation used by *Nesterenkonia* sp. strain F and *Clostridium* spp. are depicted in Fig. 4.

As shown in Table 3, a majority of the enzymes needed for solvent and acid production were active in *Nesterenkonia* sp. strain F. As indicated in Fig. 4, the pathway for acetyl-CoA production from pyruvate found in aerobic bacteria, e.g., *Nesterenkonia* sp. strain F, is different from that found in *Clostridium* spp.^35^. More specifically, in the clostridial pathway, the pyruvate resulting from glycolysis is cleaved by pyruvate-ferredoxin oxidoreductase (PFOR) in the presence of coenzyme-A to yield carbon dioxide and acetyl-CoA with the concomitant conversion of oxidized ferredoxin to its reduced form^35^. However, the formation of acetyl-CoA from pyruvate in aerobic bacteria, e.g., *Nesterenkonia* sp. strain F, is catalyzed by pyruvate dehydrogenase^35^. The pyruvate-ferredoxin oxidoreductase of Clostridia is a very unstable enzyme and very sensitive to the presence of oxygen, whereas pyruvate dehydrogenase is an active enzyme in aerobic conditions^35^. This could justify the formation of metabolites and the growth of the *Nesterenkonia* sp. strain F under aerobic conditions.

## Conclusions

Solvents, i.e., ABE, as well as acids, i.e., acetic and butyric acids, were produced simultaneously through cultivation of the bacterium *Nesterenkonia* sp. strain F under halophilic and aerobic conditions. Therefore, it was found that Clostridia are not exceptional with respect to their ability to conduct ABE fermentation. In addition, the production of solvents and acids under aerobic and halophilic conditions may make *Nesterenkonia* sp. strain F a unique bacterium for obtaining superior engineered strains for ABE production.

## Additional Information

**How to cite this article**: Amiri, H. *et al.* *Nesterenkonia* sp. strain F, a halophilic bacterium producing acetone, butanol, and ethanol under aerobic conditions. *Sci. Rep.* **6**, 18408; doi: 10.1038/srep18408 (2016).

## Figures and Tables

**Table 1:** Comparative analysis and classification of the genes encoding carbohydrate active enzymes based on the enzymes families between the *Nesterenkonia* sp. strain F and *Nesterenkonia alba* DSM 19423 genomes.

Enzyme classes	EC number	Enzyme name	Abundance overview	
			N. alba DSM 19423	N. sp. strain F
Glycoside Hydrolase family	EC:3.2.-	Hydrolases. Glycosylases.	1	1
	EC:3.2.1.1	Alpha-amylase.	0	1
	EC:3.2.1.20	Alpha-glucosidase.	0	2
	EC:3.2.1.21	Beta-glucosidase.	1	2
	EC:3.2.1.22	Alpha-galactosidase.	1	1
	EC:3.2.1.23	Beta-galactosidase.	2	2
	EC:3.2.1.24	Alpha-mannosidase.	1	0
	EC:3.2.1.52	Beta-N-acetylhexosaminidase.	1	1
	EC:3.2.1.55	Non-reducing end alpha-L-arabinofuranosidase.	2	2
	EC:3.2.1.8	Endo-1,4-beta-xylanase.	0	1
	EC:3.2.1.99	Arabinan endo-1,5-alpha-L-arabinosidase.	0	1
Glycoside Transferase family	EC:2.4.1.-	Transferases. Glycosyltransferases. Hexosyltransferases.	1	1
	EC:2.4.1.129	Peptidoglycan glycosyltransferase.	1	1
	EC:2.4.1.15	Alpha,alpha-trehalose-phosphate synthase(UDP-forming).	1	1
	EC:2.4.1.187	N-acetylglucosaminyldiphosphoundecaprenolN-acetyl-beta-D- mannosaminyltransferase.	1	0
	EC:2.4.1.227	Undecaprenyldiphospho-muramoylpentapeptidebeta-N- acetylglucosaminyltransferase.	1	1
	EC:2.4.1.250	D-inositol-3-phosphate glycosyltransferase.	1	1
	EC:2.4.1.251	GlcA-beta-(1−>2)-D-Man-alpha-(1−>3)-D-Glc-beta-(1−>4)-D-Glc-alpha-1- diphospho-ditrans,octacis-undecaprenol 4-beta-mannosyltransferase.	1	0
	EC:2.4.1.83	Dolichyl-phosphate beta-D-mannosyltransferase.	1	1
	EC:2.4.2.-	Transferases. Glycosyltransferases. Pentosyltransferases.	1	1
	EC:2.4.2.1	Purine-nucleoside phosphorylase.	1	1
	EC:2.4.2.10	Orotate phosphoribosyltransferase.	1	1
	EC:2.4.2.14	Amidophosphoribosyltransferase.	1	1
	EC:2.4.2.17	ATP phosphoribosyltransferase.	1	1
	EC:2.4.2.18	Anthranilate phosphoribosyltransferase.	1	1
	EC:2.4.2.19	Nicotinate-nucleotide diphosphorylase (carboxylating).	1	1
	EC:2.4.2.28	S-methyl-5′-thioadenosine phosphorylase.	0	1
	EC:2.4.2.4	Thymidine phosphorylase.	1	1
	EC:2.4.2.7	Adenine phosphoribosyltransferase.	0	1
	EC:2.4.2.8	Hypoxanthine phosphoribosyltransferase.	1	1
	EC:2.4.2.9	Uracil phosphoribosyltransferase.	2	2
Polysaccharide Lyase family	EC:4.2.2.2	Pectate lyase.	0	1
Carbohydrate Esterase family	EC:3.1.1.1	Carboxylesterase.	1	1
	EC:3.1.1.11	Pectinesterase.	0	1
	EC:3.1.1.17	Gluconolactonase.	0	2
	EC:3.1.1.24	3-oxoadipate enol-lactonase.	0	1
	EC:3.1.1.29	Aminoacyl-tRNA hydrolase.	1	1
	EC:3.1.1.31	6-phosphogluconolactonase.	1	1
	EC:3.1.1.45	Carboxymethylenebutenolidase.	1	1
Auxiliary Activities family	EC:1.10.3.-	Oxidoreductases. Acting on diphenols and related substances as donors. With oxygen as acceptor.	2	2
	EC:1.11.1.15	Peroxiredoxin.	1	1
	EC:1.11.1.6	Catalase.	1	1

**Table 2:** Comparative analysis and classification of the genes encoding carbohydrate active enzymes based on substrate specificity between the *Nesterenkonia* sp. strain F and *Nesterenkonia alba* DSM 19423 genomes.

Carbohydrates class	Carbohydrate active enzyme	EC number	Carbohydrate or derivates name	Abundance overview	
				N. sp. strain F	N. alba DSM 19423
Monosaccharide	EC:1.2.1.12	D-Glyceraldehyde	Glyceraldehyde-3-phosphate dehydrogenase (phosphorylating).	2	2
	EC:2.7.6.1	D-Ribose	Ribose-phosphate diphosphokinase	1	1
	EC:3.6.1.13		ADP-ribose diphosphatase	1	1
	EC:4.1.2.4		Deoxyribose-phosphate aldolase	1	1
	EC:5.3.1.6		Ribose-5-phosphate isomerase	3	2
	EC:5.3.1.4	L-Arabinose	L-arabinose isomerase	1	1
	EC:5.3.1.5	Xylose	Xylose isomerase	1	1
	EC:1.1.1.22	D-Glucose	UDP-glucose 6-dehydrogenase	1	1
	EC:1.1.1.49		Glucose-6-phosphate dehydrogenase (NADP(+))	1	1
	EC:2.3.1.157		Glucosamine-1-phosphate N-acetyltransferase	1	1
	EC:2.4.1.15		Alpha,alpha-trehalose-phosphate synthase (UDP-forming)	1	1
	EC:2.4.1.187		N-acetylglucosaminyldiphosphoundecaprenol N-acetyl-beta-D- mannosaminyltransferase	0	1
	EC:2.4.1.227		Undecaprenyldiphospho-muramoylpentapeptide beta-N- acetylglucosaminyltransferase	1	1
	EC:2.5.1.7		UDP-N-acetylglucosamine 1-carboxyvinyltransferase	1	1
	EC:2.7.1.63		Polyphosphate–glucose phosphotransferase	1	1
	EC:2.7.7.12		UDP-glucose–hexose-1-phosphate uridylyltransferase	1	1
	EC:2.7.7.23		UDP-N-acetylglucosamine diphosphorylase	1	1
	EC:2.7.7.9		UTP–glucose-1-phosphate uridylyltransferase	1	1
	EC:2.7.8.33		UDP-N-acetylglucosamine–undecaprenyl-phosphate N-acetylglucosaminephosphotransferase	1	1
	EC:3.5.1.25		N-acetylglucosamine-6-phosphate deacetylase	1	0
	EC:3.5.99.6		Glucosamine-6-phosphate deaminase.	1	0
	EC:5.1.3.14		UDP-N-acetylglucosamine 2-epimerase (non-hydrolyzing)	1	0
	EC:5.1.3.2		UDP-glucose 4-epimerase	1	1
	EC:5.3.1.9		Glucose-6-phosphate isomerase	1	1
	EC:5.4.2.10		Phosphoglucosamine mutase	1	1
	EC:1.1.1.336	D-Mannose	UDP-N-acetyl-D-mannosamine dehydrogenase	1	0
	EC:2.4.1.187		N-acetylglucosaminyldiphosphoundecaprenol N-acetyl-beta-D- mannosaminyltransferase	0	1
	EC:2.4.1.251		GlcA-beta-(1−>2)-D-Man-alpha-(1−>3)-D-Glc-beta-(1−>4)-D-Glc-alpha-1- diphospho-ditrans,octacis-undecaprenol 4-beta-mannosyltransferase.	0	1
	EC:2.4.1.83		Dolichyl-phosphate beta-D-mannosyltransferase	1	1
	EC:2.7.7.13		Mannose-1-phosphate guanylyltransferase	1	1
	EC:5.3.1.8		Mannose-6-phosphate isomerase	1	1
	EC:2.7.1.29	Glycerone	Glycerone kinase	3	1
	EC:5.1.3.1	D-Ribulose	Ribulose-phosphate 3-epimerase	1	1
	EC:5.1.3.4		L-ribulose-5-phosphate 4-epimerase	1	1
	EC:1.1.1.267	D-Xylulose	1-deoxy-D-xylulose-5-phosphate reductoisomerase	1	1
	EC:2.2.1.7		1-deoxy-D-xylulose-5-phosphate synthase	1	1
	EC:2.6.1.16	D-Fructose	Glutamine–fructose-6-phosphate transaminase (isomerizing)	1	1
	EC:3.1.3.11		Fructose-bisphosphatase	1	1
	EC:4.1.2.13		Fructose-bisphosphate aldolase	1	1
Oligosaccharides & Polysaccharides	EC:3.2.1.1	starch, glycogen	Alpha-amylase	1	0
	EC:3.2.1.20	Oligosaccharides	Alpha-glucosidase (Maltase)	2	0
	EC:3.2.1.21	Oligosaccharides	Beta-glucosidase (Cellobiase)	2	1
	EC:3.2.1.22	galactose oligosaccharides, galactomannans and galactolipids	Alpha-galactosidase	1	1
	EC:3.2.1.23	beta-D-galactosides	Beta-galactosidase (lactozym)	2	2
	EC:3.2.1.24	alpha-D-mannosides	Alpha-mannosidase	0	1

**Table 3:**
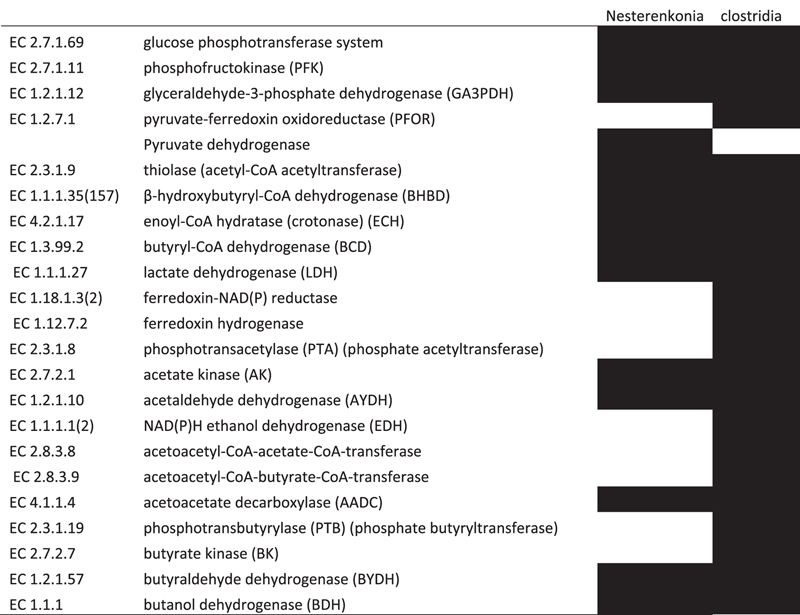
Comparative analysis of the genes encoding active enzymes pertaining to ABE fermentation between the *Nesterenkonia* sp. strain F and solvent-producing Clostrida, i.e., *C. acetobutylicum* and *C. beijerinckii*.
